# Detection of POLE Subtypes in High-Grade Endometrioid Carcinoma by BaseScope-ISH Assay

**DOI:** 10.3389/fonc.2019.00831

**Published:** 2019-09-04

**Authors:** Shuangni Yu, Huilin Shao, Xinchao Ban, Hongkai Zhang, Yan You, Na Zhou, Xinxin Mao, He Zhao, Jie Chen, Zhaohui Lu

**Affiliations:** ^1^Department of Pathology, Peking Union Medical College Hospital, Chinese Academy of Medical Sciences, Peking Union Medical College, Beijing, China; ^2^Department of Pathology, Beijing Hospital of Traditional Chinese Medicine, Capital Medical University, Beijing, China; ^3^Department of Oncology, Peking Union Medical College Hospital, Chinese Academy of Medical Sciences, Peking Union Medical College, Beijing, China

**Keywords:** POLE, endometrioid carcinoma, detection, BaseScope-ISH, P286R, V411L

## Abstract

**Objective:** The identification of DNA polymerase epsilon (POLE) mutation subtypes in endometrial cancer is critical for molecular classification. The mutation of the POLE gene could only be detected by sequencing until now. We propose to validate and develop the feasibility of using BaseScope, an *in situ* hybridization (ISH) assay, for the detection of POLE mutations in high-grade endometrioid carcinomas (EC).

**Methods:** Among 51 paraffin-embedded samples of high-grade EC, BaseScope-ISH assays were used to detect the RNA mutation status of the POLE gene, mainly focusing on two hotspot mutations of P286R and V411L. The number of positive signals in the cytoplasm was counted, setting the positive threshold and determining the *in situ* hybridization results. The sensitivity and specificity of BaseScope-ISH assay were compared with that of the Sanger sequencing results.

**Results:** Based on the BaseScope assay, there were 19 positive samples and 32 negative samples in a total of 51 samples. Of the 19 positive samples, 10 samples showed P286R site mutations in the POLE gene, while the other nine samples were V411L site mutations. Only one sample with the V411L site mutation identified by Sanger sequencing showed negative signal value. The remaining 31 cases without the P286R site mutation or V411L site mutations all showed negative signal. This analysis result showed the sensitivity was 95% and the specificity was 100% for the BaseScope assay detecting POLE mutants in high-grade EC.

**Conclusion:** In the case of high-grade EC, combined with morphological characteristics, the BaseScope assay can effectively and specifically identify POLE mutation cases, providing a reliable foundation for the application of clinical diagnosis and molecular classification.

## Introduction

Endometrial cancer is one of the most common gynecologic malignanciesin developed countries (1). In China, it ranks second in female reproductive system malignant tumors. According to the National Cancer Center, the incidence rate in 2015 was 0.643‰, and the mortality rate was 0.218‰ in China. Recently, the incidence of endometrial cancer has increased (2). Some gene alterations facilitating endometrial tumorigenesis have been identified (3–6). The Cancer Genome Atlas Research Network (TCGA) has divided endometrial carcinomas into four categories according to various genetic and epigenetic features: an ultramutator phenotype caused by DNA polymerase epsilon (POLE) mutations, a hypermutator phenotype caused by DNA mismatch repair deficiency (dMMR) leading to microsatellite instability (MSI), a copy number low phenotype, and a copy number high phenotype (3).

POLE, the catalytic subunit of DNA polymerase ε, is implicated in nuclear DNA replication and repair (7). Most studies show that the most common mutations of POLE were located in the exonuclease domain (exons 9–14), which are involved in proofreading activity during DNA replication. These mutations caused an unusually high mutational burden, leading to a hypermutated phenotype in colorectal and endometrial carcinomas (8). Endometrioid carcinoma (EC) is the primary subtype of endometrial carcinoma, accounting for 70–80% of cases. In EC patients, the most common mutations in POLE were in exon 9 (P286R and S297F) and exon 13 (V411L, L424V, and L424I), in which two mutations (P286R and V411L) accounted for 63–76% of all POLE point mutations. It has been established that POLE mutations like these contribute to a better prognosis and longer progression-free survival (3, 4, 9–11).

Meanwhile, POLE mutant cases have received much more attention because their morphological feature is inconsistent with the prognostic outcome. Currently, for EC patients, most tailored treatments recommended are based on the histological type, stage, and grade (12–15). However, it has been recently found that the efficacy of molecular typing in judging prognosis exceeds the histomorphological diagnosis to a certain extent, especially for high-grade endometrial carcinomas, which have significant overlaps in histopathologic and immunohistochemical characteristics (9, 16–19).

Molecular typing of endometrial cancer is of great significance for guiding clinical treatment and judging the prognosis of patients. Currently, this subtype is mainly recognized by conventional Sanger sequencing, and there are no other methods or alternatives for molecule detection used in clinical diagnosis. In our study, we will use specific BaseScope mutation probes to identify POLE mutations, and analyze the sensitivity and specificity of the BaseScope-ISH assay in ECs.

## Materials and Methods

### Human Samples

The information from “high-grade endometrioid carcinoma” cases was recorded in the archives of the Beijing Union Medical College Hospital from June 1, 2010, to November 30, 2017. A total of 202 cases were retrieved, and there were 161 cases with paraffin-embedded tumor tissues available. Fifty-one cases were selected for our studies, including 39 POLE gene (exon 9–14) mutation samples and 12 samples containing no POLE exon 9–14 mutations. All cases were under ethical approval (PUMCH committee, No. S-K688), and both informed and written consents were obtained from all patients of this study when they were admitted to the hospital (the written consents were kept in archives with every patient's files). The average time since the fixation of tissue blocks was 4 years (range 0–8 years). At least three expert histopathologists examined samples.

### Histotype and Grade Assignment

We had original diagnoses from the archived data on both diagnostic (curettage specimens) and final hysterectomy specimens. Also, two gynecologic subspecialty pathologists (Zhaohui Lu and Shuangni Yu) reviewed hematoxylin and eosin stained slides from diagnostic and final hysterectomy specimens with the goal of assigning histotype and grade. Only those specimens of grade 3 or 2–3 and endometrioid carcinoma were kept in the study. These pathologists were blind to the original pathology reports and to each other's interpretations.

### TMA Construction

For all diagnostic endometrioid carcinoma samples (endometrial curettage and hysterectomy specimens), two pieces of tissue microarrays were constructed using 2 mm cores per case in duplicate at random.

### Sanger Sequencing

DNA extracted using the QiaAMP DNA micro kit (QIAGEN Ltd, Manchester, UK) was used for polymerase chain reaction (PCR) to amplify POLE exons 9–14. PCR primers were designed (Table 1), and PCR products (150–200 bp) were amplified with the input of 100 ng FFPE derived DNA. Sequencing was performed using BigDye v3.1 terminator cycle sequencing chemistry on the ABI 3730 DNA analyzer (Applied Biosystems Inc., Foster City, CA). All validated POLE mutations were bidirectionally sequenced twice using Sanger sequencing.

**Table 1:** Primers of sequencing.

**POLE Exon**	**Primer sequence**	
Exon 9	Forward	TCTTTTAACAACCAGAGGGAGGT
	Reverse	TTGCTCCCATTCCTGGACTAA
Exon 10	Forward	CACATTGCTGTGGACTTCTTTG
	Reverse	CTCATGGAGCTGCAATTCTGA
Exon 11	Forward	ATGAGGCTGCTGCTTCTGAAC
	Reverse	CCAGGAGCCACCTCCTAAGTC
Exon 12	Forward	ACTCTGCACCTCCCGTGTCT
	Reverse	TCCCATGAGATGTGGTGACAG
Exon 13	Forward	GCCCAGTTTTGCCAGTTCT
	Reverse	GAGCGGGCTGGCATACAT
Exon 14	Forward	CTGTGCCGGTCTCCTTACTGT
	Reverse	GGGACATCCACCTCCATTCAG

### BaseScope Assay

BaseScope assays were performed and assisted following guidelines (BaseScope™ Detection Reagent Kit-RED User Manual) provided by the supplier (Advanced Cell Diagnostics, Newark, CA). Briefly, sections were cut at 4 μm thickness onto Superfrost plus slides (Thermo Scientific, New Hampshire, USA) and allowed to dry overnight at room temperature (RT). Sections were then baked at 60°C for one h before being deparaffinized in xylene (2 ×5 min) and ethanol (2 ×2 min), then dried by baking at 60°C for 2 min. Pretreat 1 (hydrogen peroxide) was applied for 10 min at RT, Pretreat 2 (target retrieval) for 15 min at 100°C and Pretreat 3 (protease IV) for 30 min (tissue sections) at 40°C, with two rinses in distilled water between pretreatments. BaseScope probes (four types of candidate probes were all purchased from Advanced Cell Diagnostics) (see Table 2) were then applied for 2 h at 40°C in a HybEZ oven before incubation with reagents AMP0 (30 min at 40°C), AMP1 (15 min at 40°C), AMP2 (30 min at 40°C), AMP3 (30 min at 40°C), AMP4 (15 min at 40°C), AMP5 (30 min at RT), and AMP6 (15 min at RT). Slides were rinsed with wash buffer (2 ×2 min) between AMP incubations. Finally, slides were incubated with Fast Red for 10 min at room temperature in the dark. Then, slides were counterstained with hematoxylin before drying for 15 min at 60°C.

**Table 2:** Details of BaseScope probes.

**Probe type**	**Target amino acid variant**	**Target nucleotide variant**
Negative control-dapB	N/A	N/A
Positive control-POLR2A	N/A	N/A
POLE P286 mutant	P286R	857C > G
POLE V411 mutant	V411L	1231G > C/T

### Signal Quantification and Statistical Analysis

The signals were observed and counted by eye under the microscopy (at 400 × magnification) as a preliminary investigation and digital images of signals were photographed for the convenience of display and a second look. The hybridized images were scanned into digital data, the cytoplasm signals were recorded, and statistical analysis was made for each sample (400 × High magnification vision field). The average value was calculated, and the cutoff value was set at 31 red dots signal/3.14 mm^2^ (≈10/mm^2^), positive samples: ≥31 red dots signal/3.14 mm^2^; negative samples: <31 red dots signal/3.14 mm^2^. All data were represented in mean ± standard deviation (SD), and statistical significance was set at *p* <0.05 (two-tailed). We also calculated the sensitivity, specificity, positive predictive value (PPV), and negative predictive value (NPV). Statistical analyses were performed using Student's *t*-test.

## Results

### Validation of BaseScope Probes in High-Grade EC

**Figure 1:**
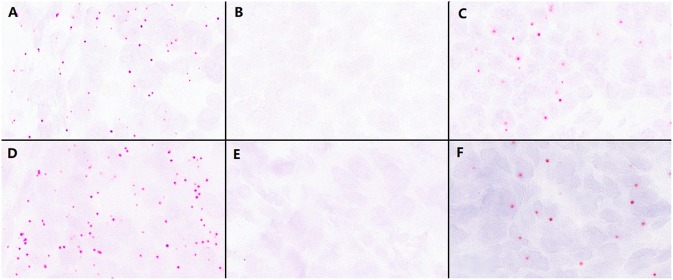
Validation of the effectiveness of the BaseScope probe in tumor tissues. **(A)** The analysis of RNA quality by the P286R positive control probes in P286R mutated high-grade endometrioid carcinoma (EC), showing a large number of positive signals. **(B)** Negative control probes showed no positive signal in P286R mutated EC. **(C)** The detection of P286R mutations in high-grade EC by BaseScope *in situ* hybridization, showing multiple strong red signals in the cytoplasm. **(D)** The analysis of RNA quality by V411L positive control probes in V411L mutated high-grade EC, showing a large number of positive signals. **(E)** Negative control probes showed no positive signal in V411L mutated EC. **(F)** The detection of V411L mutation in high-grade EC by BaseScope *in situ* hybridization, showing multiple strong red signals in the cytoplasm; Scale bars represent 60 microns.

First, we used the positive control probe to detect RNA status in paraffin-embedded high-grade EC samples, and the results showed numerous strong positive hybridization signals (Figures 1A,D); then we used two types of BaseScope probes, one specific for the P286R point mutation (857C > G) and the other for the V411L point mutation (1231G > C/T), respectively, to test high-grade EC samples by ISH. The results showed positive staining signals (Figures 1C,F), but little to no signal in the negative samples (<31 positive signals/1 core of tissue array, or 10 positive signals/mm^2^) (Figures 1B,E).

### POLE Mutation Sequencing and Sensitivity and Specificity of the BaseScope Assay

We sequenced POLE gene exons 9–14 in 161 high-grade EC samples from our center, and the results showed 39 patients possessed point mutations in their POLE gene exons 9–14. By reverse sequencing, the results confirmed that these 39 samples included mutations in the POLE exon region, 10 cases with the P286R site mutation, and 10 with the V411L site mutation, which comprised 51% of all the mutations.

We made tissue microarrays using 51 cases of high-grade EC, including the 10 cases of the P286R mutation and 10 cases of the V411L mutation. Twenty cases with other point mutations in POLE exons 9–14 (including one case with P286R mutation and another point mutation of the POLE exons 9–14), and 12 cases without POLE exon 9–14 mutations were used as controls.

**Figure 2:**
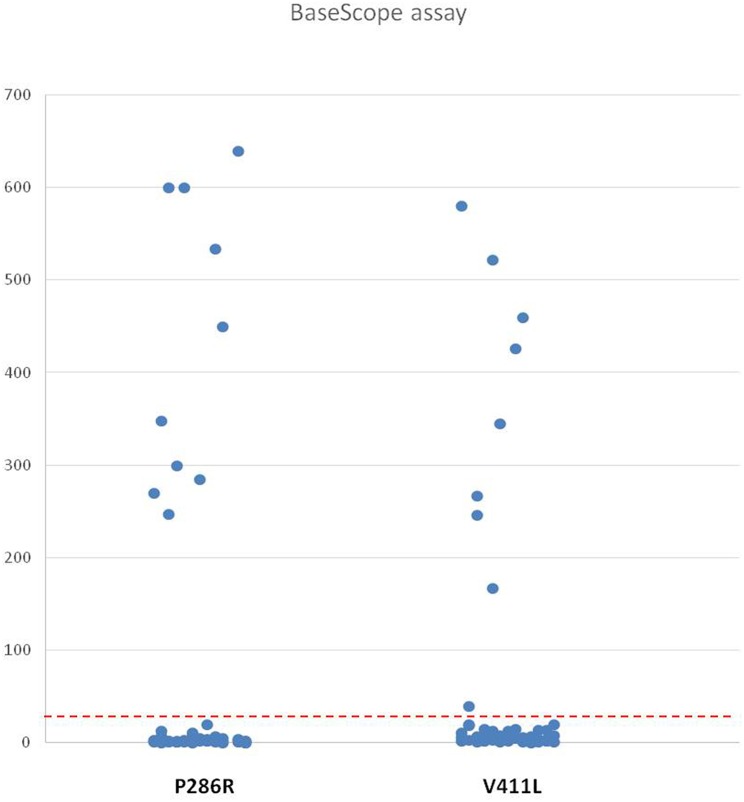
The Number of the Positive Signal of Two Probes in High-Grade EC. The ordinate shows the value of the positive signal of P286R and V411L probes in high-grade EC microarrays (51 cases), and the red dashed line represents the positive threshold (31/3.14 mm^2^).

The number of positive signal points of the BaseScope-ISH in each tissue microarray was collected; they are shown in Table 3, ranging between 0 and 640/3.14 mm^2^. The positive threshold was set to 31/3.14 mm^2^ (10/mm^2^). BaseScope-ISH results showed that 19 of 51 cases were positive based on the threshold (Figure 2). Ten of 19 positive samples were P286R point mutations, and nine cases were V411L site mutations, which is entirely consistent with sequencing results. In the BaseScope-ISH negative samples, only one case in which a V411L site mutation was identified by sequencing showed a few signal points (3/3.14 mm^2^), which was below the positive threshold and inconsistent with sequencing results. The remaining cases were all negative, the same as the sequencing results. There was a significant difference in the number of signal points between the P286R probe positive group (mean ± standard deviation: 427 ± 155) and the negative group (mean ± standard deviation: 3 ± 4) (*P* <0.001). The number of signal points in the V411L probe positive group (mean ± standard deviation: 339 ± 176) and the negative group (mean ± standard deviation: 6 ± 6) were also significantly different (*P* <0.001). For the sensitivity and specificity of BaseScope detection, the P286R probe was 100% (10 samples were all detected); the sensitivity of the V411L probe was 90% (only 1 of 10 samples was not detected), and the specificity was 100%. The positive and negative predictive value of the P286R site mutation in the POLE gene were both 100%; for the V411L site mutation, they were 90 and 97.6%, respectively.

**Table 3:** Number of positive signals in high-grade endometrioid carcinoma tissues (mean value of two tissue microarray core per case, each tissue microarray core area is about 3.14 mm^2^).

**Sample number**	**Age of block (months)**	**No. of signal by Basescope (P286R)**	**No. of signal by Basescope (V411L)**	**Mutational status by sequencing (Sanger), exon 9–14 of POLE**	**Results of RNA-ISH**
1	92	3	12	NM^*^	–
2	90	2	20	NM	–
3	57	248	7	P286R mutation	+
4	57	300	15	P286R mutation	+
5	48	2	14	NM	–
6	44	7	345	V411L mutation	+
7	42	5	7	NM	–
8	39	4	5	NM	–
9	38	2	460	V411L mutation	+
10	36	450	5	P286R mutation	+
11	36	4	14	NM	–
12	36	2	14	NM	–
13	32	270	20	P286R mutation	+
14	30	14	580	V411L mutation	+
15	29	600	19	P286R mutation	+
16	23	2.5	267	V411L mutation	+
17	17	600	9	P286R mutation	+
18	17	12	168	V411L mutation	+
19	16	285	8	P286R mutation	+
20	15	20	13	NM	–
21	13	8	15	NM	–
22	7	6	7	NM	–
23	16	1	1	NM	–
24	89	1	1	NM	–
25	85	2	2	NM	–
26^‡^	84	348	2	P286R mutation	+
27	82	2	3	NM	–
28	76	1	40	V411L mutation	+
29	72	1	2	NM	–
30	68	1	3	NM	–
31	57	2	3	V411L mutation	–^§^
32	55	3	3	NM	–
33	51	2	2	NM	–
34	50	1	426	V411L mutation	+
35	47	640	3	P286R mutation	+
36	44	1	7	NM	–
37	44	4	5	NM	–
38	44	7	5	NM	–
39	39	1	8	NM	–
40	36	2	7	NM	–
41	24	3	3	NM	–
42	24	0	246	V411L mutation	+
43	21	3	2	NM	–
44	17	2	522	V411L mutation	+
45	17	534	1	P286R mutation	+
46	13	2	3	NM	–
47	8	3	6	NM	–
48	6	3	2	NM	–
49	5	1	1	NM	–
50	5	3	3	NM	–
51	4	1	2	NM	–

NM*: no mutation in POLE Exon 9–14.

‡ *Including the site 12,250 mutations located in Exon 11*.

§ *V411L mutation was not detected by Basescope-ISH*.

### Comparison of Mixed Probes to Single Probes

**Figure 3:**
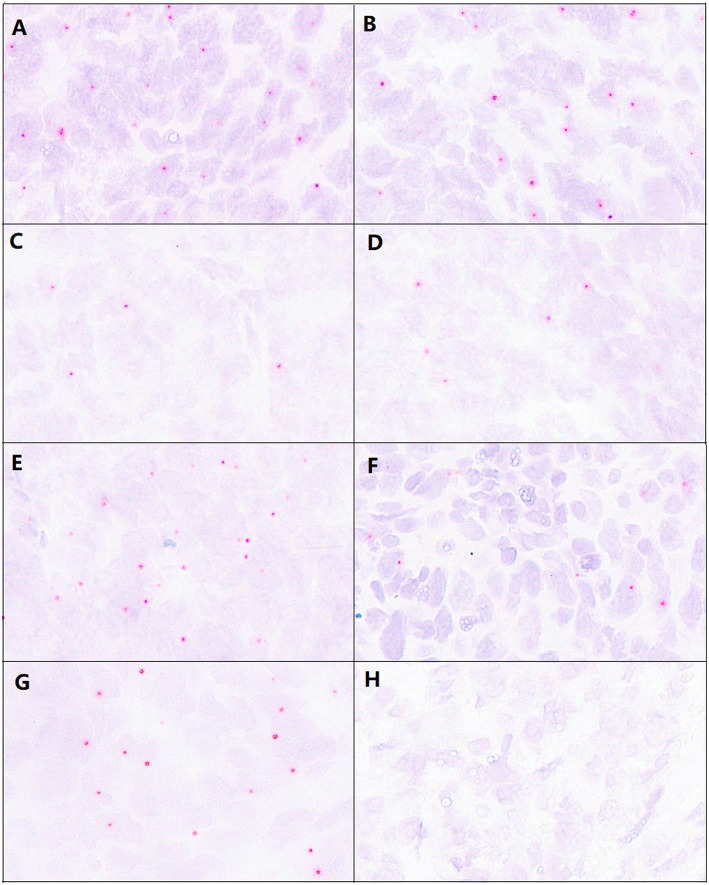
Comparison of detection results from mixed and single probes and paraffin section signal images before and after 5 months. **(A)** The signal of the P286R probe in P286R mutated high-grade EC. **(B)** The signal of mixed probes (P286R and V411L probe) in P286R mutated high-grade EC. **(C)** The signal of the V411L probe in V411L mutated high-grade EC. **(D)** The signal of mixed probes (P286R and V411L probe) in V411L mutated high-grade EC. **(E,F)** The detection using the P286R probe in P286R high-grade EC paraffin section before **(E)** and after **(F)** 5 months, the hybridization signal counts were significantly reduced 5 months after in paraffin sections. **(G,H)** The detection using the V411L probe in V411L high-grade EC paraffin section before **(E)** and after **(F)** 5 months, the hybridization signal counts were significantly reduced 5 months after in paraffin sections.

In order to detect the two mutation sites simultaneously, we used two probe mixtures to hybridize 51 high-grade EC samples and found 19 mutations samples, including 10 cases of P286R point mutations, and nine cases of V411L point mutations. The case involving the V411L mutation identified by sequencing but negative by the single probe still did not reach the positive threshold (the number of positive signals is 17/3.14 mm^2^)using mixed probes. The result of mixed probes was utterly consistent with the single probe detection with a sensitivity of 95% and a specificity of 100% (Figures 3A–D).

### Comparison of Slide Detection at 5 Months and After

We prepared one tissue microarray with 22 tumor tissue samples, including seven cases of the P286R site mutation, five cases of the V411L site mutation, six cases of other mutations within POLE exons 9–14, and four cases without exon 9–14 mutations in the POLE gene. We placed these sample slices at room temperature for 5 months and then repeated BaseScope-ISH.

**Figure 4:**
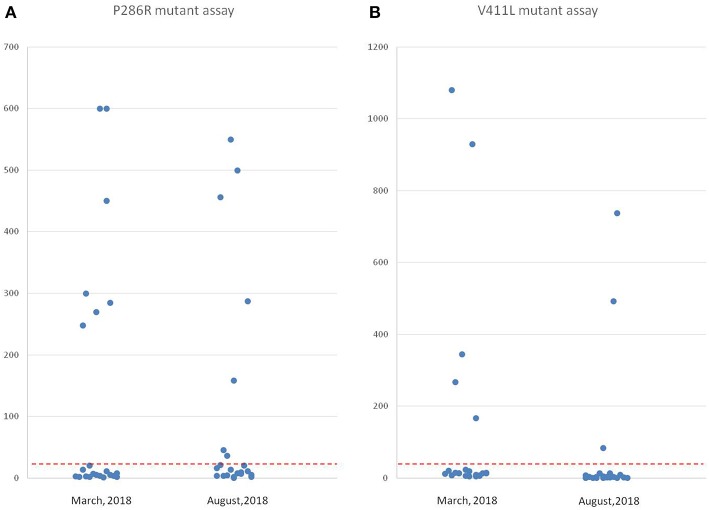
Comparison of point mutation signals detected by *in situ* hybridization 5 months before and after in paraffin sections using two probes. **(A)** The values of the positive signal of POLE P286R mutations from the same group of high-grade EC tissues at two time points (22 cases). The left side is the result from March 2018, and the right side is the result from August 2018 (the number of positive signals decreased). **(B)** The values of the positive signal of POLE V411L mutations from the same group of high-grade EC tissues at two-time points (22 cases). The left side is the result from March 2018, and the right side is the result from August 2018 (the number of positive signals decreased remarkably, and the signal values of two cases fell below the threshold).

For the samples with P286R site mutations in their POLE gene, the number of positive signal points decreased (Figures 3E,F), but the number of positive samples was still 7, and the positive detection rate had no change (100%). In the POLE gene V411L point mutation samples, the number of positive signal points was decreased (Figures 3G,H), the number of signals in two samples did not reach the positive threshold, and the positive detection rate decreased by 40%. However, the specificity of these two probe detections was still perfect (100%) (Figure 4).

## Discussion

BaseScope-ISH is an effective, highly sensitive, and specific method for detecting point mutations in RNA level (20). It has been reported to be used in studying tumor heterogeneity in colon cancer tumor tissues (21). Some researchers have successfully detected the ALK and PD-L1 gene mutations in lung cancer (22, 23). In our study, we used the BaseScope probes of two hot POLE gene mutations to test the high-grade EC samples, which surmounted the disadvantages of screening mutant samples by sequencing. It is more useful for the samples with less tissue because it does not require additional tissue for DNA extraction. Meanwhile, we could observe and count the positive signals under microscopy by eye when tumor cell morphology was directly combined as reference. Assessment of signals by eye provided acceptably high sensitivity and specificity, which would allow this methodology to be more widely used in clinical practice. Equally important we performed signal interpretation by intuitively and closely combining tumor cell morphology. The positive signals were counted only when they were within the cytoplasm or nucleus of tumor cells. The bulky nuclei of tumor cells were outlined by hematoxylin. Signals presenting in normal endometrium, vascular wall, and fibrotic or necrotic tissues were interpreted as false negative signals and wouldn't be included. This combination of signal reading with cell morphology would bring us an accurate evaluation of assay.

The individualized treatment of tumors is closely related with the molecular typing of tumor genes (24–26), and tumor with the POLE gene mutation, representing a unique subtype of EC, owns a quite better prognosis. In particular, these tumors often appear to be high-grade. And in the absence of the knowledge of their POLE gene mutation status, they would be mistakenly included into a category of tumors with much worse prognosis than they actually have. So, the identification of this mutated subgroup is essential for better personalized treatment and prognosis analysis of endometrial cancer (3, 17), and our findings made a vital step toward this goal.

The POLE gene mutation rate in high-grade EC is 10–26% (9, 16, 17, 27), and the mutation rate in our center's samples was 24% (39/161 cases), which is consistent with the reported mutation rate. There are many different kinds of point mutations in the POLE gene, which are mainly distributed in the exon 9–14 region. The most common mutations are at site 857 (amino acid 286, P286R) and 1231 (amino acid 411, V411L), accounting for 63–76% of all mutation types (3, 27, 28). The mutation rate of these two hotspots in our samples was 51% of the entire exon 9–14 region mutations. Compared with the literature, the ratio was slightly low. We found that the mutation site in our samples distribution spectrum was wide—which might be a characteristic of disease-related mutations in our Chinese population.

Our study also showed that two mutation points could be detected at the same time if we use mixed probes to test samples, with over half of the mutations recognized in one test. This significantly improves the efficiency of mutation case screening, and has great significance for clinical diagnosis. Further studies would be carried out to look for the probes that are able to recognize the other identifiable mutations, as well as to answer the questions about how many such probes could be effectively combined.

There are two subtypes of mutated bases for the POLE gene V411L (1231G > C/T) site mutation (4, 9, 27): G/C and G/T. In 10 cases of the V411L mutation samples we tested, five cases were G-mutated to C, and five cases were G-mutated to T. We detected these two mutations simultaneously when using BaseScope-ISH, indicating that this probe can recognize two mutations at the same site. In practical applications, it will remarkably increase the detection types and detection efficiency. For POLE gene V411L mutation detection, the number of positive signals in most mutant samples was much higher than the threshold. However, in one sample, the number of positive signals was 40, slightly higher than the positive threshold (31 signals); and the number in another sample did not reach the positive threshold. This may be caused by the small proportion of mutant cells or sample degradation before fixation. The possibility of uneven distribution caused by tumor heterogeneity is less likely because all of the samples were randomly acquired, and signals were detected in all other mutant samples. So, for the majority of samples, the tissue microarray is considered to be a good representative.

The POLE mutations can be detected in archived paraffin-embedded samples(The earliest sample used in this study was collected in 2010). However, for paraffin tissue slices placed at room temperature for 5 months, some RNA degradation may occur, resulting in a significantly reduced detection rate, and the omission of some positive cases. Therefore, it is no longer suitable for RNA *in situ* hybridization detection to be used on long-term, preserved slices stored at room temperature. In daily work, we should pay attention to sample preservation, and use freshly prepared paraffin slices for ISH detection of RNA point mutations when possible.

## Conclusion

BaseScope-ISH is a significant advancement and improvement in screening paraffin-embedded EC tissue samples for POLE mutations, which can enhance the identification of POLE mutation subtypes and provide a powerful strategy for the molecular classification and detection of other tumors involving known gene mutations.

## Data Availability

The datasets analyzed in this manuscript are not publicly available. Requests to access the datasets should be directed to luzhh@pumch.cn.

## Author Contributions

ZL: experimental design, supervision, and results analysis. SY and HS: experiments, manuscript writing, and data analysis. XB: sample collection and data analysis. HKZ and JC: critical reading and revision of the manuscript. YY and XM: evaluation of positive signals. NZ: figure and table construction. HZ: preparation of tissue slice and technical supports.

### Conflict of Interest Statement

The authors declare that the research was conducted in the absence of any commercial or financial relationships that could be construed as a potential conflict of interest.
